# A LASSO-derived risk model for long-term mortality in Chinese patients with acute coronary syndrome

**DOI:** 10.1186/s12967-020-02319-7

**Published:** 2020-04-06

**Authors:** Yi-ming Li, Zhuo-lun Li, Fei Chen, Qi Liu, Yong Peng, Mao Chen

**Affiliations:** 1 Department of Cardiology, West China Hospital, Sichuan University, 37 Guoxue Street, Chengdu, 610041 China; 2 Department of Computer Science and Engineering, Tandon School of Engineering, New York University, New York, USA

**Keywords:** Acute coronary syndrome, Risk model, Machine learning, LASSO, Random forest

## Abstract

**Background:**

The formal risk assessment is essential in the management of acute coronary syndrome (ACS). In this study, we develop a risk model for the prediction of 3-year mortality for Chinese ACS patients with machine learning algorithms.

**Methods:**

A total of 2174 consecutive patients who underwent angiography with ACS were enrolled. The missing data among baseline characteristics were imputed using the MissForest algorithm based on random forest method. In model development, a least absolute shrinkage and selection operator (LASSO) derived Cox regression with internal tenfold cross-validation was used to identify the predictors for 3-year mortality. The clinical performance was assessed with decision curve analysis.

**Results:**

The average follow-up period was 27.82 ± 13.73 months; during the 3 years of follow up, 193 patients died (mortality rate 8.88%). The Kaplan–Meier estimate of 3-year mortality was 0.91 (95% confidence interval (CI): 0.890.92). After feature selection, 6 predictors were identified: Age,” “Creatinine,” “Hemoglobin,” “Platelets,” “aspartate transaminase (AST)” and “left ventricular ejection fraction (LVEF)”. At tenfold internal validation, our risk model performed well in both discrimination (area under curve (AUC) of receiver operating characteristic (ROC) analysis was 0.768) and calibration (calibration slope was approximately 0.711). As a comparison, the AUC and calibration slope were 0.701 and 0.203 in Global Registry of Acute Coronary Events (GRACE) risk score, respectively. Additionally, the highest net benefit of our model within the entire range of threshold probability for clinical intervention by decision curve analysis demonstrated the superiority of it in daily practice.

**Conclusion:**

Our study developed a prediction model for 3-year morality in Chinese ACS patients. The methods of missing data imputation and model derivation base on machine learning algorithms improved the ability of prediction. .

*Trial registration* ChiCTR, ChiCTR-OOC-17010433. Registered 17 February 2017–Retrospectively registered

## Introduction

As the unstable and progressive stage of coronary heart disease (CHD), acute coronary syndrome (ACS) includes three serious and life-threating clinical manifestations: ST-segment elevation myocardial infarction (STEMI), non-STEMI, and unstable angina pectoris [1, 2]. The prognosis of ACS patients varies considerably for different pathophysiological changes in individuals based on their level of disease. Thus, a formal assessment to identify high-risk patients is essential in the management of ACS [3].

Currently, the Global Registry of Acute Coronary Events (GRACE) is the most commonly used risk assessment tool and is recommended by guidelines for predicting short- and long-term mortality [4]. However, the GRACE risk score was developed in North America, South America, and Europe but included few participants in Asia [5]. The clinical performance of this risk score has not been assessed in the Chinese population. A risk tool derived from Clinical Pathways for Acute4 Coronary Syndromes (CPACS) investigators for Chinese patients with ACS has been described previously [6]. However, this CPACS risk score only predicted hospital mortality and did not use algorithms to avoid overfitting in the model estimation. Therefore, we aim to develop a specific risk model for the prediction of long-term (3-year) mortality for Chinese ACS patients in a hospital-based dataset.

## Results

### Study population and outcomes

From January 2009 to September 2012, a total of 2174 ACS patients were included in this study. The average follow-up period was 27.82 ± 13.73 months; during the 3 years of follow up, 193 patients died (mortality rate 8.88%), including 121 cases of cardiac death (cardiac mortality was 5.57%) and 55 cases of non-cardiovascular death. 31 patients were categorized into unknown death. The Kaplan–Meier estimate of 3-year mortality was 0.91 (95% confidence interval (CI) 0.890.92). The baseline characteristics of this study population were stratified according to patients who survived until the end of the follow-up period and those who did not survive. The mean age of the patients was 64.54 ± 10.57 years, and the number of male patients was 1713 (78.79%). More than half of patients had hypertension (1183, 54.64%) and one in five had diabetes (470, 21.72%) or previous myocardial infarction (379, 23.9%). Meanwhile, the higher percentage of usage of evidence-based medications were found in survival group, including aspirin, clopidogrel, beta-blockers, angiotensin-converting-enzyme inhibitors or angiotensin II receptor blockers and statins. The difference between two groups is summarized in Table 1.

**Table 1:** Clinical characteristics of the study population

Characteristics	Total	Patients survived	Patients died	*P* Value
No. of patients	N = 2174	N = 1981	N = 193	
Age	64.54 ± 10.57	63.96 ± 10.54	70.54 ± 8.83	<0.001
Gender, man, n (%)	1713 (78.79%)	1572 (79.35)	141 (73.06)	0.041
Medical history				
Pre-hypertension, n (%)	1183 (54.64)	1072 (54.31)	111 (58.12)	0.313
Pre-diabetes mellitus, n (%)	470 (21.72)	412 (20.88)	58 (30.37)	0.002
Pre-heart failure, n (%)	83 (5.3)	60 (4.3)	23 (12.2)	<0.001
Pre-myocardial infarction, n (%)	379 (23.9)	329 (23.6)	50 (26.3)	0.475
At admission				
HR, beats/min	74.99 ± 26.09	74.51 ± 26.69	79.96 ± 18.05	<0.001
SBP, mm Hg	130.51 ± 22.1	130.68 ± 21.52	128.79 ± 27.43	0.263
DBP, mm Hg	76.29 ± 12.8	76.52 ± 12.64	73.88 ± 14.11	<0.001
LVEF, %	50.52 ± 24.62	50.55 ± 24.95	50.22 ± 19.41	0.896
Risk assessment				
GRACE risk score	92.95 ± 26.1	91.35 ± 25.64	109.44 ± 25.2	<0.001
TIMI classification	4.58 ± 2.03	4.39 ± 1.94	5.94 ± 2.20	<0.001
Laboratory values				
Serum creatinine, μmol/L	94.44 ± 51.3	92.69 ± 47.87	112.37 ± 76	<0.001
Blood glucose, mmol/L	7.17 ± 3.3	7.03 ± 3.11	8.59 ± 4.6	<0.001
Total cholesterol, mmol/L	4.13 ± 1.11	4.13 ± 1.1	4.09 ± 1.2	0.602
WBC, n × 10^9^/L	7.92 ± 5.66	7.79 ± 5.75	9.23 ± 4.43	<0.001
RBC, n × 10^12^/L	4.45 ± 1.11	4.47 ± 1.15	4.16 ± 0.65	<0.001
Hemoglobin, g/L	134.22 ± 35.39	135.17 ± 36.42	124.42 ± 19.89	<0.001
Platelets, n × 10^9^/L	162.28 ± 61.05	161.79 ± 60.75	167.33 ± 63.95	0.229
AST, U/L	56.91 ± 102.95	52.55 ± 81.82	102.8 ± 222.7	<0.001
Serum K^+^, mmol/L	3.96 ± 0.47	3.96 ± 0.45	4 ± 0.62	0.261
Serum Ca^2+^, mmol/L	2.32 ± 4.4	2.34 ± 4.61	2.16 ± 0.18	0.619
Fibrinogen, g/L	3.83 ± 11.22	3.8 ± 11.46	4.18 ± 8.23	0.679
Discharge medications				
Aspirin, n (%)	2005 (94.18%)	1865 (96.68%)	140 (70.0%)	<0.001
Clopidogrel, n (%)	2002 (94.03%)	1854 (96.11%)	148 (74.0%)	<0.001
ACEI/ARBs, n (%)	1228 (57.73%)	1140 (59.16%)	88 (44.0%)	<0.001
Beta-blockers, n (%)	1434 (67.45%)	1340 (69.57%)	94 (47.0%)	<0.001
Statins, n (%)	1949 (91.55%)	1807 (93.68%)	142 (71.0%)	<0.001

Data are expressed as mean ± SD or counts and percentages, as appropriate

*HR* heart rate, *SBP* systolic blood pressures, *DBP* diastolic blood pressure, *LVEF* left ventricular ejection fraction, *GRACE* Global Registry of Acute Coronary Events, *TIMI* thrombolysis in myocardial infarction, *WBC* white blood cell, *RBC* red blood cell, *AST* aspartate transaminase, *ACEI* angiotensin-converting-enzyme inhibitors, *ARB* angiotensin II receptor blockers

The Killip classifications were excluded as predictors in the model because of a large amount of missing data and the difficulty of conducting accurate measurements. The details of missing data among baseline characteristics are listed in Additional file 1.

### Model derivation

**Fig. 1:**
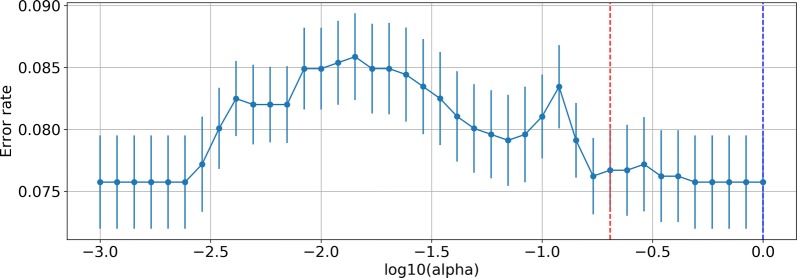
10-fold cross-validated error plot: The blue dot line equals lambda with the minimum error, whereas the red dot line is the lambda we manually choose

First, we conducted Cox regression with the least absolute shrinkage and selection operator (LASSO) penalization to perform predictor selection, which can help reduce the dimensions of a prediction model. To determine the penalty factor (lambda), a tenfold cross-validated error plot of the lasso model was constructed as shown in Fig. 1. The optimal lambda was determined by choosing the most regularized and parsimonious model within 1 standard error from the minimum.

**Fig. 2:**
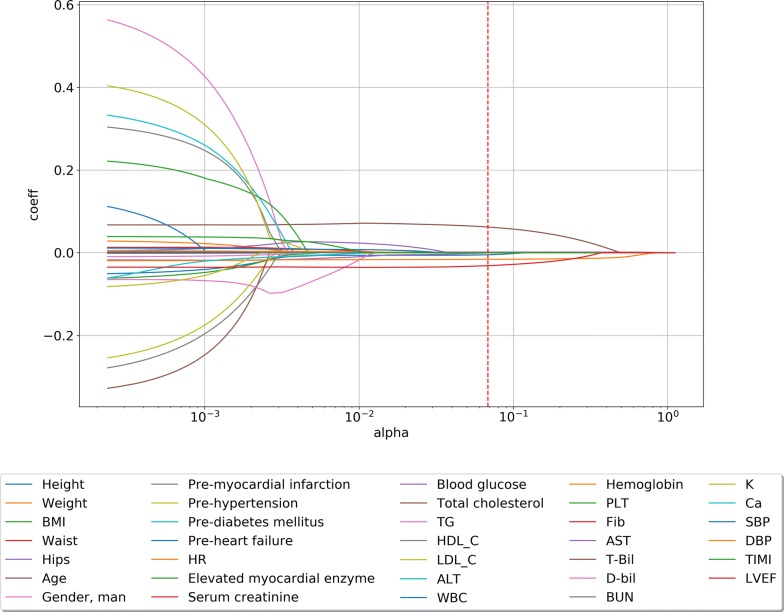
LASSO path of all coefficients of predictors at varying log-transformed lambda values: The red dot line is the lambda we manually choose. *LASSO* least absolute shrinkage and selection operator, *BMI* body mass index, *HR* heart rate, *SBP* systolic blood pressures, *DBP* diastolic blood pressure, *LVEF* left ventricular ejection fraction, *WBC* white blood cell, *RBC* red blood cell, *AST* aspartate transaminase, *ALT* alanine transaminase, *BUN* blood urea nitrogen, *T-Bil* total bilirubin, *D-Bil* direct bilirubin, *HDL-C* high-density lipoprotein cholesterol, *LDL-C* low density lipoprotein cholesterol, *TG* triglyceride, *PLT* platelets, *Fib* Fibrinogen, *TIMI* thrombolysis in myocardial infarction

Because of the imbalance in our data, even the most parsimonious model with 0 characters was less than 7.7%, and that model was also within 1 standard error. To balance the power of the lasso penalty and the accuracy of our model, after some experiments with different lambdas, we manually choose a proper lambda that is still within 1 standard error and provided good results. The lambda is shown in Fig. 1. The LASSO path of all coefficients of predictors at varying log-transformed lambda values is shown in Fig. 2. We added the thrombolysis in myocardial infarction (TIMI) classification in predictor selection just as reference.

The final LASSO model with the optimal lambda included the following 6 non-zero variables: “Age,” “Serum creatinine,” “Hemoglobin,” “Platelets,” “aspartate transaminase (AST),” and “left ventricular ejection fraction (LVEF)”. After we determined the most important predictors, the prediction model was developed using normal Cox regression without penalization. Under the proportional hazard assumption, the baseline hazard function can be estimated by Breslow’s Estimator. The formula for predicting the risk of 3-year mortality is as follows:

$$\begin{aligned} H(t = 36\left| {x_{\alpha } } \right.) = \exp (Age*0.7504017 + Serum\;creatinine*0.00166143 + Haemoglobin* - 0.01728725 + Platelets*0.00154873 + AST*0.0013414 + LVEF* - 0.03612834)*H_{0} (36) \end{aligned}$$

where $H_{0\,} \,(36)\, = \,0.02727.$ We created an Excel file of this formula to favor workability in daily practice. Additionally, the use of this predictive model was demonstrated in 5 patients from this study population (Additional file 2).

### Model validation

**Fig. 3:**
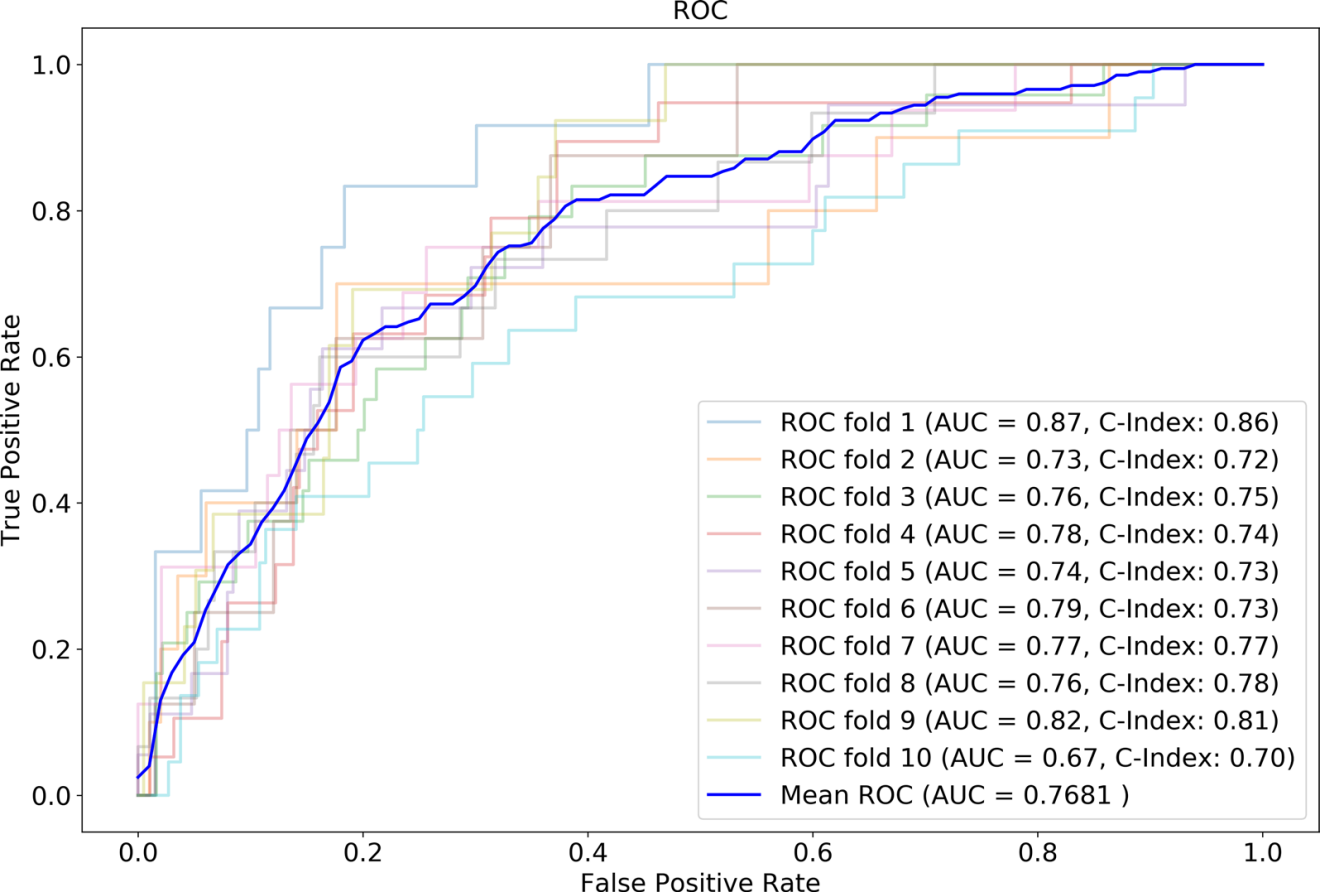
Tenfold cross-validation and ROC analysis result of our model for the prediction of 3-year mortality. *ROC* receiver operating characteristic curve

**Fig. 4:**
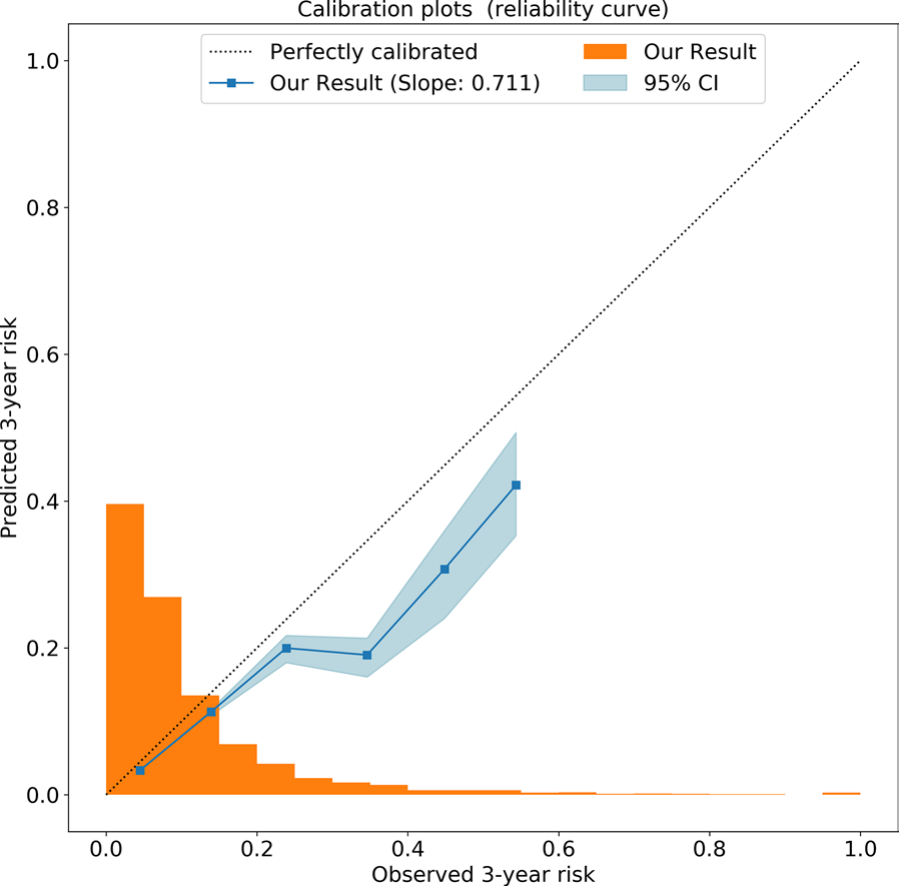
Calibration plot: Calibration plot showing the agreement between predicted (x-axis) and observed (y-axis) 3-year risk of the mortality. Squares represent binned Kaplan–Meier estimates with 95% confidence filled with the blue area. The dotted line represents perfect calibration. The bar histogram on the x-axis reflects the percentage of patients with a predicted risk corresponding to the x-axis

The discrimination by tenfold cross-validation and receiver operating characteristic curve (ROC) analysis result of our risk model for the prediction of 3-year mortality was good (area under curve (AUC) = 0.7681) (Fig. 3). However, the GRACE score had relatively poor power in the discriminative ability (AUC = 0.709). The Harrell’s C-statistic was 0.7601 for this risk model. We found good agreement between the predicted and observed 3-year risk of mortality. The calibration slope was approximately 0.711, as calculated by linear least-squares regression of the given points in the calibration plot (Fig. 4). The calibration slope for the GRACE score was 0.203. Thus, our model exhibited better calibration than the GRACE score.

### Clinical performance

**Fig. 5:**
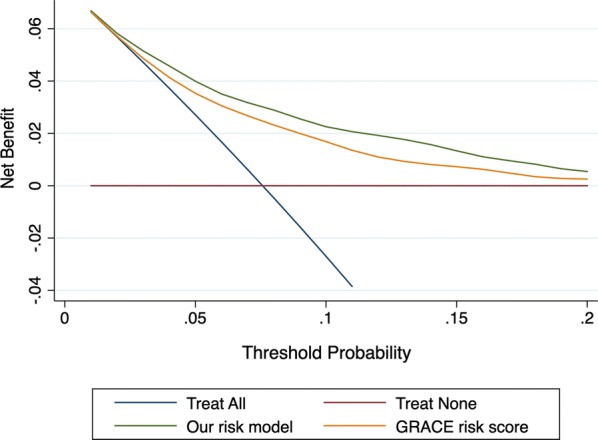
Decision curve analysis: Decision curve analysis comparing the clinical performance of our risk model (the green line) and the GRACE risk score (the yellow line). For risk of 3-year mortality, our risk model showed the highest net benefit for all potential thresholds (ranging from 0% to 20%). This demonstrated that our model would result in the highest weighted balance of clinical intervention for ACS patients, regardless of the risk threshold. ACS: acute coronary syndrome, GRACE: Global Registry of Acute Coronary Events

We performed a decision curve analysis to compare the clinical utility of our risk model and the GRACE score. Because all of the treatments for ACS, including percutaneous coronary intervention (PCI) and thrombolysis, involve some harm for patients, the optimal decision threshold was > 0%. We observed the highest net benefit of our model within the entire range of threshold probability for clinical intervention (Fig. 5). This indicates the superiority of our risk model in clinical performance, regardless of the risk threshold for PCI or thrombolysis.

## Discussion

In this study, we developed a risk model to predict the long-term mortality in Chinese ACS patients and performed internal validation of this model. Compared to the GRACE risk score, our risk model demonstrated better discriminative ability, improved calibration and a greater net benefit for clinical performance. Furthermore, we used machine learning methods such as random forest imputation and a penalty algorithm to maintain statistical power and avoid overfitting during model derivation. To the best of our knowledge, this is the first prediction model for long-term mortality in Chinese ACS patients.

The predictors selected by this risk model include “Age,” “Creatinine,” “Hemoglobin,” “Platelets,” “AST,” and “LVEF”. These risk factors could be supported by existing theories and research. Usually, older patients are more fragile and have more comorbidities. Many studies have considered age to be an independent predictor of ACS, and in studies focusing on other risk factors, age usually needs to be adjusted [7]. Creatinine or eGFR levels are thought to be associated with mid- and long-term mortality in ACS patients, and ACS patients with renal insufficiency are more likely to experience bleeding and other complications when given invasive treatment [8, 9]. In previous studies, baseline hemoglobin levels or anemia status were predictors of 30-day and 1-year mortality in patients with ACS or STEMI [10], while hemoglobin levels of 1416 g/dl resulted in the lowest risk of death [11]. Studies have reported that AST is associated with microvascular obstruction in ACS patients, and its predictive value is even better than that of NT-proBNP [12]. A meta-analysis of 8 studies indicated that high baseline platelet levels would increase short-term and long-term mortality in ACS patients [13], which may be related to the pathological basis of coronary heart disease involving the platelet-granulocytic system and acute pathogenesis of ACS involving intravascular inflammatory mechanisms [14]. Finally, the LVEF is considered as a marker of cardiac function in heart disease, and the guidelines also recommend ultrasound or angiography for NST-ACS patients to evaluate left ventricular function [3]. Low baseline LVEF is a predictor of mortality and MACE in ACS patients [15].

The GRACE risk score was developed based on 123 hospitals in 14 countries but only involved a small number of Chinese patients [4]. Most of the related studies on risk assessment of Chinese ACS patients investigated the domestic optimization or application of GRACE risk score. Previous CPACS studies have only reported patients with in-hospital mortality [6]. Therefore, there is no long-term risk prediction tool for the Chinese ACS population. This study is the first attempt for this purpose, and several new machine learning algorithms were used to improve the accuracy of the model.

This prediction model was established according to the Transparent Reporting of a Multivariable Prediction Model for Individual Prognosis or Diagnosis (TRIPOD) statement [16], and we also referred to the opinion of ABCD proposed by Ewout W for validation [17]. Additionally, we did not use the conventional multiple imputations for missing value processing but applied a novel random forest algorithm. The random forest algorithm has been demonstrated as an efficient method to handle missing data. It can manage different types of missing data and can scale to high dimensions [18]. Several different imputation algorithms based on random forests have been developed, and among them, MissForest was found to have a noticeable improvement on performance compared to other methods such as the k-nearest neighbors and parametric MICE methods [19]. In this dataset, random forest imputation had a higher statistical power and better accuracy for prediction than complete-case analyses (AUC of the ROC for tenfold cross-validation, 0.744).

In the model derivation, we used the LASSO-Cox method to estimate the relationship between predictors and time-event. LASSO regularization is a method to manage overfitting and perform variable selection and has been widely used many types of machine learning algorithms [20]. It adds the L1 norm of coefficients as the penalty term to the loss function and hence adds constraints to the coefficients. In contrast to ridge regularization, LASSO regularization performs different degrees of shrinkage on variables and pushes some coefficients to zero. When adding the LASSO method to the Cox model, the estimation variance is reduced, and a subset of predictors is selected while providing an interpretable Cox model [21]. To ensure the accuracy of the model, we did not use a nomogram to simplify the parameters in the model presentation but to estimate the patient’s death risk through the cumulative hazard using the Cox model. This model showed good consistency (AUC of the ROC for tenfold cross-validation and C-statistic) for patients who died within 3 years and good agreement (slope and plot) for the actual and predicted 3-year mortality risk. The clinical usefulness of this model mainly lies in its ability to quantify the long-term mortality of patients by combining baseline data before angiography at an individual level. DCA could be used to evaluate whether our model is more advantageous for clinical applications than the GRACE model, which is currently widely used in clinical research [22]. This method could help physician to assess the value of information provided by a risk assessment tool or test by weighted the potential risk and benefit [23]. For all risk thresholds > 0%, our model showed a higher net benefit than the GRACE model. Therefore, we believe that our model can better help patients understand the disease and help doctors make clinical decisions. Particularly for patients with a high risk of ACS, doctors can use this model to assess whether patients can benefit from treatment.

There were some limitations of our study. First, the present study lacked external validation. In addition, due to the number of samples, there were relatively few death events in this dataset. However, careful statistical methods were used for the machine learning and penalty algorithms to ensure the accuracy of the model and prevent overfitting.

## Conclusion

Our study developed a prediction model based on machine learning for 3-year morality in Chinese ACS patients. The external validation and further studies are needed to confirm the usefulness of it.

## Methods

### Study population

The data source for this investigation was the West China Hospital CHD database. This single center database prospectively includes all CHD or high-risk patients undergoing angiography in West China Hospital affiliated to Sichuan University. For this analysis, we enrolled consecutive CHD patients from January 2009 to September 2012 who were included in the database. ACS patients were eligible for inclusion if they had (1) angiographic evidence of ≥ 50% stenosis in ≥ 1 coronary vessel; (2) ischemic chest discomfort that increased or occurred at rest; and/or (3) electrocardiography or cardiac biomarker criteria consistent with ACS. The exclusion criteria were malignancies, pregnancy, end stage renal disease and severe liver or hematological diseases. These inclusion and exclusion criteria were met by 2406 continuous CHD patients enrolled from the database. After excluding patients with loss of follow-up (n = 192) and much missing data (n = 40), 2174 patients were included in the data analysis. The study protocol was approved by the local institutional review boards in accordance with the Declaration of Helsinki. All subjects provided written informed consent before enrolment.

### Baseline characteristics

Demographic data, medical history, cardiovascular risk factors, vital signs at admission, medication at discharge, and the final diagnosis were obtained from the patients’ electronic medical records and reviewed by a trained study coordinator. Blood samples were collected at admission and before angiography, and plasma biomarkers including Fib, liver and kidney function, blood glucose, and serum lipids were analyzed in the Department of Laboratory Medicine, West China Hospital, accredited by the College of American Pathologists. The Elevated myocardia enzyme is defined as the cardiac troponin T or Creatine kinase-MB raised beyond the upper limit of laboratory reference values. Hypertension was defined as systolic blood pressure (SBP) ≥ 140 mm Hg, diastolic blood pressure (DBP) ≥ 90 mm Hg and/or patients receiving antihypertensive medications. Diabetes mellitus was diagnosed in patients who had previously undergone dietary treatment for diabetes, had received additional oral antidiabetic or insulin medications or had a current fasting blood glucose level of ≥ 7.0 mmol/L or a random blood glucose level ≥ 11.1 mmol/L. The GRACE risk prediction tool used for analysis of mortality has been described previously [4]. The calculation of the GRACE risk score was performed using an online program (http://www.outcomes-umassmed.org/grace).

### Follow-up and study outcome

The follow-up period ended in January 2013. Follow-up information was collected through contact with the patients’ physicians, patients or their family. All data were corroborated with the hospital records. The primary endpoint of this study was all-cause mortality, and the secondary endpoint was cardiovascular death, as documented in the database. Death was considered to be cardiac death when it was caused by acute myocardial infarction (MI), significant arrhythmias, or refractory heart failure. Sudden unexpected death occurring without another explanation was considered cardiovascular death.

### Statistical analyses

Baseline demographics and clinical characteristics were compared between non-surviving patients and survivors. Continuous variables are expressed as the mean ± standard deviation (SD), and categorical variables are reported as counts and percentages. T-tests and Chi squared tests were used to evaluate differences between groups for continuous and categorical variables, respectively. The Kaplan–Meier method was used to calculate the rate of cumulative events during the follow-up period.

### Missing data

To avoid loss of statistical power, all missing data among baseline characteristics were assumed to be missing at random and imputed using a random Forest-based imputation method [18, 24]. Specifically, the “MissForest” method was used. MissForest handles missing data by iteratively using Random Forests. It starts by imputing the missing values of the candidate column, which is the column with the least missing values. Then, the imputer fills other missing values of the remaining columns with a mean imputation and uses them as predictors to perform a random Forest model with the candidate column as output. The missing values of the candidate column are imputed according to the prediction made from the fitted random Forest. This process starts over again for the remaining columns and repeats over multiple times until a certain stopping criterion is met.

### Model derivation and validation

The development and validation of this risk model followed the Transparent Reporting of a Multivariable Prediction Model for Individual Prognosis or Diagnosis (TRIPOD) statement [16]. The independent predictors of 3-year mortality were identified among baseline characteristics using a Cox proportional-hazards regression model. The proportional hazard assumption was verified using the Schoenfeld residuals method.

When performing model estimation, the LASSO method was applied to avoid the overfitting, and the penalty parameter was selected by cross-validation [20, 21]. According to design of our study, only the clinical characteristic before intervention were put into LASSO path and feature selection. The LASSO method is a shrinkage regression technique using L1 regularization and designed for high-dimensional data. Furthermore, this algorithm shrinks the coefficients of noninfluential predictors to zero and thus excludes them from the final model. This technique has been widely used in both machine learning and clinical practice. The estimated risk of mortality of a given patient was calculated from the cumulative hazard function of the Cox model as follows:

$$H\,(t\left| {x_{\alpha } } \right.)=\,\exp \,(x_{\alpha }^{T} \,\beta )\,H_{0} \,(t).$$

In this equation, $H_{0\,} \,(t)$ is the baseline hazard function of time t, and $x_{\alpha }^{T} \,\beta$ is the linear product of the predictors and associated coefficients for a patient.

The model was validated with tenfold cross-validation [25]. In the assessment of the discrimination ability of the prediction model, Harrell’s C-statistic was used to estimate the degree of discrimination, and ROC analysis was conducted for visual inspection. Furthermore, the calibration was investigated using a calibration plot by plotting the predicted and observed probabilities of events across increasing levels of predicted risk.

### Clinical performance

To assess the utility of our model in clinical practice, we compared this risk model to the GRACE score. First, we sought to examine the difference in the AUC of ROC between these two risk-assessment tools. Second, we performed decision-curve analysis to quantify the clinical usefulness of our prediction model (which was also compared with that of the GRACE score) [26]. This analysis was used to assess the net true-positive classification rate using a model over a range of thresholds. The values (from 0 to 1) represent the benefit from clinical intervention, and higher values indicate more significant benefit.

Data analyses were performed using Python (version 3.6) with the scientific libraries “scikit-learn”, “scikit-survival”, “lifelines” and Stata Statistical Software (Release 15. College Station, TX: StataCorp LLC).

## Supplementary information

**Additional file 1:** The details of missing data among variables.

**Additional file 2:** The risk formula of our model and the demo of use it.

## Data Availability

The datasets used and/or analysed during the current study are available from the corresponding author on reasonable request.

## Abbreviations

CHD: Coronary heart disease
ACS: Acute coronary syndrome
STEMI: ST-Segment elevation myocardial infarction
GRACE: Global Registry of Acute Coronary Events
CPACS: Clinical pathways for acute coronary syndromes
HR: Heart rate
SBP: Systolic blood pressures,
DBP: Diastolic blood pressure
SD: Standard deviation
TRIPOD: Transparent reporting of a multivariable prediction model for individual prognosis or diagnosis
LASSO: Least Absolute Shrinkage And Selection Operator
ROC: Receiver operating characteristic curve
AUC: Area under curve
CI: Confidence interval
LVEF: Left ventricular ejection fraction
TIMI: Thrombolysis in myocardial infarction
WBC: White blood cell
RBC: Red blood cell
AST: Aspartate transaminase
PCI: Percutaneous coronary intervention
ACEI: Angiotensin-converting-enzyme inhibitors,
ARB: Angiotensin II receptor blockers
