# Supplementary material for: A LASSO-derived risk model for long-term mortality in Chinese patients with acute coronary syndrome
Source: J Transl Med. 2020 Apr 6;18:157. doi: 10.1186/s12967-020-02319-7 (PMC7137217; doi:10.1186/s12967-020-02319-7)
Supplement: Supplementary file 1 — Additional file 1: The details of missing data among variables. [file 12967_2020_2319_MOESM1_ESM.docx]

Additional file 1. The details of missing data among variables

| Variables | Missing n, % |
| --- | --- |
| Age | 3, 0.1% |
| Height | 0, 0% |
| Weight | 0, 0% |
| Waist | 72, 3.3% |
| Hips | 75, 3.4% |
| BMI | 6, 0.3% |
| Pre-hypertension | 9, 0.4% |
| Pre-diabetes mellitus | 10, 0.5% |
| Pre-heart failure | 18, 0.8% |
| Pre-myocardial infarction | 0, 0% |
| HR | 21, 1.0% |
| SBP | 30, 1.4% |
| LVEF | 569, 26.2% |
| Serum creatinine | 10, 0.5% |
| Blood glucose | 7, 0.3% |
| Total cholesterol | 0, 0% |
| WBC | 10, 0.5% |
| RBC | 0, 0% |
| Hemoglobin | 0, 0% |
| Platelets | 0, 0% |
| AST | 113, 5.2% |
| ALT | 4, 0.2% |
| Serum K^+^ | 2, 0.1% |
| Serum Ca^2+^ | 140, 6.4% |
| Fibrinogen | 191, 8.8% |
| TG | 0, 0% |
| HDL_C | 0, 0% |
| LDL_C | 0, 0% |
| T-Bil | 113, 5.2% |
| D-Bil | 114, 5.2% |
| BUN | 110, 5.1% |

Abbreviations: HR: heart rate, SBP: systolic blood pressures, DBP: diastolic blood pressure, LVEF: left ventricular ejection fraction, WBC: white blood cell, RBC: red blood cell, AST: aspartate transaminase, ALT: alanine transaminase, BUN: blood urea nitrogen, T-Bil: total bilirubin, D-Bil: direct bilirubin, HDL-C: High-density lipoprotein cholesterol, LDL-C: Low density lipoprotein cholesterol, TG: Triglyceride
